# Safety of Intrathecal Clonidine as an Adjuvant to Spinal Anesthesia in Infants and Children

**DOI:** 10.1111/pan.15091

**Published:** 2025-03-03

**Authors:** Grant Heydinger, Eden E. Bayer, Catherine Roth, Sibelle Aurelie Yemele Kitio, V. Rama Jayanthi, Arlyne Thung, Joseph D. Tobias, Giorgio Veneziano

**Affiliations:** ^1^ Department of Anesthesiology and Pain Medicine Nationwide Children's Hospital Columbus Ohio USA; ^2^ Department of Anesthesiology and Pain Medicine The Ohio State University College of Medicine Columbus Ohio USA; ^3^ The Ohio State University College of Medicine Columbus Ohio USA; ^4^ Department of Urology Nationwide Children's Hospital Columbus Ohio USA; ^5^ Frank H. Netter School of Medicine Quinnipiac University Hamden Connecticut USA; ^6^ Department of Anesthesiology St. Vincent's Medical Center Bridgeport Connecticut USA

**Keywords:** intrathecal clonidine, pediatric anesthesiology, spinal anesthesia

## Abstract

**Introduction:**

Preliminary clinical studies have demonstrated that clonidine is an effective adjuvant to spinal anesthesia in neonates and infants. However, the studies conducted previously have had a limited cohort size of 80–100, potentially limiting an accurate measure of its safety.

**Methods:**

The current study retrospectively examines our 5–6‐year experience with clonidine as an adjuvant to spinal anesthesia in a large cohort of neonates and infants.

**Results:**

The study cohort included 1420 patients ranging in age from newborn to 36 months (median age 7 months). Ninety‐five percent of the patients tolerated spinal anesthesia without requiring conversion to general anesthesia, and over 73% of the patients did not require any additional intraoperative sedation. Hypotension (sBP ≤ 60 mmHg) was the most common intraoperative event (17%) with one patient requiring the administration of an anticholinergic agent for bradycardia. No serious intraoperative adverse events were noted. Post Anesthesia Care Unit (PACU) Phase I was bypassed in 75% of cases, and the postoperative admission rate was 7%, with the majority (85%) being planned admissions. Fifty‐six patients (4%) returned to the hospital during the first seven postoperative days, primarily for surgical concerns.

**Conclusions:**

Based on this retrospective, observational study, clonidine appears to be a safe adjuvant to spinal anesthesia for ambulatory surgical procedures in infants and children. We observed a low incidence of intraoperative and postoperative complications.

## 1 Introduction

Clonidine is an α_2_‐adrenergic agonist originally applied clinically for the treatment of hypertension [1]. More recently, it has been shown to be a safe and effective adjuvant for neuraxial anesthesia in adults, especially within obstetrical practice [2, 3]. However, there is less understanding regarding its use for neuraxial anesthesia in infants and children. Spinal anesthesia has been shown to be a safe and effective alternative to general anesthesia (GA) for certain surgical procedures. This may be particularly beneficial in pediatric populations, as it avoids the need for airway manipulation and obviates concerns parents may have related to the perceived risks of general anesthesia [4, 5]. An impediment to the use of spinal anesthesia in infants is the short duration of action relative to adults. Clonidine has been shown to prolong the duration of spinal anesthesia as well as reduce the intraoperative requirements for sedation and analgesics. Regarding its safety profile, Walker et al. previously demonstrated that intrathecal clonidine did not produce signs of spinal cord toxicity in rat pups, even at super‐therapeutic dosages [6]. While preliminary clinical studies in humans have also indicated that clonidine is safe to use as an intrathecal adjuvant in spinal anesthesia, these cohorts have generally been limited to fewer than 100 patients [7, 8]. Although adverse effects including bradycardia, hypotension, and respiratory depression have been reported, the smaller cohort size may limit an accurate evaluation of the safety and adverse effect profile of clonidine [9, 10, 11].

We previously reported our experience with spinal anesthesia instead of GA in more than 1200 pediatric patients, demonstrating the safety and effectiveness of spinal anesthesia as an anesthetic technique, but we did not differentiate between patients that did or did not receive clonidine. Since that time, we have continued to successfully administer intrathecal clonidine to hundreds of infants undergoing spinal anesthesia [4], and we present our experience in the present manuscript.

## 2 Methods

### 2.1 Study Design and Cohort

This study is a retrospective chart review focused on pediatric patients who received spinal anesthesia instead of general anesthesia from 2016 until July 2022 for general surgical and urological procedures. The study was approved by the Institutional Review Board at Nationwide Children's Hospital. As a retrospective study, the need for individual patient consent was waived. Patients were identified through a search of the operating room schedule and electronic medical records using the keywords “regional anesthesia” and “0.5% bupivacaine” in intraoperative documentation or a procedural note indicating spinal anesthesia was performed. Patients who received intrathecal clonidine in addition to bupivacaine for spinal anesthesia were identified and included in the study cohort.

### 2.2 Technique for Spinal Anesthesia

Spinal anesthesia was administered either by an attending pediatric anesthesiologist or under the supervision of the Acute Pain Service & Regional Anesthesia Team at Nationwide Children's Hospital. The procedure adhered to our standardized protocol for spinal anesthesia, which involves the application of 4% topical lidocaine cream to the lumbar spine area 30 min prior to the procedure [4, 5]. In the operating room, a regional anesthesia timeout was conducted. The skin over the lumbar spine was disinfected with chlorhexidine, and the patient was positioned sitting with neck flexion and lumbar extension. A 22‐gauge, 1.5‐in. Quincke spinal needle was used for the lumbar puncture (LP) performed at the L3‐L4 or L4‐L5 vertebral level. Upon entering the intrathecal space and confirmation of cerebrospinal fluid flow, the local anesthetic agent was injected. The anesthetic agent typically consisted of 1 mg/kg (up of 1.2 mL) of 0.5% isobaric bupivacaine with epinephrine 1:200000 and 1 μg/kg of clonidine. Our clinical practice is to prepare the spinal solution ourselves in the operating room just prior to administration. Plain bupivacaine is used as the base solution, and epinephrine as well as 100 μg/mL clonidine is added using a 1 mL volume syringe. Depending on the clinical need, an epinephrine “wash” technique was used, involving drawing and then expelling undiluted epinephrine (1 mg/mL), leaving only a trace amount inside the syringe before filling it with the local anesthetic solution and clonidine. Following the intrathecal injection of the anesthetic agent, the patient was placed in the supine position, and intravenous (IV) access was secured in a lower extremity after sensory blockade was confirmed. A surgical timeout followed the establishment of IV access prior to the start of the surgical procedure. If the patient remained agitated despite adequate spinal blockade, 24% sucrose on a pacifier was used for soothing. If this did not control the agitation and the patient was unable to be soothed, IV dexmedetomidine (0.5–1 μg/kg) was administered. Because dexmedetomidine is highly selective for α_2_‐adrenergic receptors, we find that it achieves an effective level of mild sedation without major hemodynamic effects. This is preferred over opioids, as analgesia is not necessary with a working spinal blockade. There is also less risk of respiratory depression compared to benzodiazepines or propofol. Following surgery, patients were moved directly to Phase II of the post‐anesthesia care unit (PACU) if they did not require supplementary IV sedation, or Phase I for monitoring and assessment if they received additional sedation during the procedure. A modified Aldrete scoring system was used to evaluate readiness for transition to phase 2 [12, 13]. Scores range from 0 to 10 based on points assigned for respiration, color, activity, circulation, and consciousness. A score ≥ 8 or a return to clinical baseline indicated readiness for Phase 2 recovery, where patients were reunited with their caregivers or guardians and prepared for subsequent discharge. Patients that were born at full term (> 37 weeks) were candidates for same‐day discharge at 44 weeks postconceptual age, and patients born preterm (< 37 weeks) could be discharged home if > 60 weeks postconceptual age on the day of surgery.

### 2.3 Data Collection

Data collected for the study included patient demographic data (age, height, weight, gender, ethnicity, race, and American Society of Anesthesiologists [ASA] physical status), anesthesia details (dose of local anesthetic, level of LP for spinal anesthesia, number of LP attempts, time for placement of spinal anesthesia, time to surgical incision following placement of spinal anesthesia, need for supplemental sedation, conversion to GA, intraoperative medications, postoperative medications), surgical details (type of procedure, duration of procedure, intraoperative hemodynamic or respiratory adverse events), and recovery details (length of PACU stay, need for supplemental oxygen, time to oral feed, discharge time, ED or UC return within 7 days).

The primary outcome was the overall safety of clonidine as an adjuvant to the medication regimen for spinal anesthesia. Safety outcomes were evaluated by the identification of adverse effects related to spinal anesthesia or IT clonidine specifically. These included respiratory effects, including the need for supplemental oxygen, oxygen desaturation defined as saturation ≤ 92%, apnea, bradypnea, or the need for bag‐valve‐mask ventilation. Adverse hemodynamic effects included bradycardia defined as heart rate ≤ 80 beats per minute, hypotension defined as systolic blood pressure (sBP) ≤ 60 mmHg, or the clinical need for the administration of fluid boluses or vasoactive/anticholinergic medications.

### 2.4 Statistical Analysis

Demographic and intraoperative characteristics were analyzed using descriptive statistics. Categorical data were summarized as frequencies and percentages. Continuous data, which exhibited skewed distributions as assessed by the Shapiro–Wilk test, were presented as medians with interquartile ranges. All statistical analyses were conducted using Stata software (version 16; StataCorp, College Station, TX).

## 3 Results

The study cohort included 1420 patients ranging in age from newborn to 36 months, with the majority (99%) of patients being less than 18 months of age. The median age was 7 months (IQR 6, 10 months). Ninety‐eight percent of the patients were males. Ninety‐six percent of the patients were assigned ASA physical classification I or II (Table 1). Isobaric bupivacaine was used most often as the local anesthetic agent (98%). Epinephrine was added to the spinal anesthetic solution in 96% of the procedures, and all patients received intrathecal clonidine (Table 2). Procedures included a mix of urologic and general surgery procedures. Lumbar puncture and spinal anesthesia failed in 76 patients (5.3%), who required conversion to GA, mostly due to an incomplete motor block. Three‐hundred and seventy‐eight patients (26.6%) received additional sedation intraoperatively; Table 3 highlights additional sedation medications given.

**TABLE 1:** Demographic characteristics of study population.

Variables	N (%)	Median (IQR)
Study Population	1420	
Age (months)		7 (6, 10)
Gender		
Male	1386 (97.6)	
Female	34 (2.4)	
Weight (kg)		8.5 (7.5, 9.5)
Height (cm)		69 (65.4, 72.5)
Ethnicity		
Hispanic	32 (2.3)	
Non‐Hispanic	1348 (94.9)	
Unknown	40 (2.8)	
Race		
Asian	28 (2.0)	
Black/African American	193 (13.6)	
White	1088 (76.6)	
Other	89 (6.3)	
Unknown	22 (1.5)	
ASA Classification		
I	948 (66.8)	
II	420 (29.6)	
III	49 (3.4)	
IV	3 (0.2)	

*Note:* Data presented as: Count (percentages) and median (interquartile range).

Abbreviation: ASA, American Society of Anesthesiologists.

**TABLE 2:** Procedure and anesthetic characteristics of study population.

Variables	*N* (%)	Median (IQR)
Study Population	1420	
Total procedure time (minutes)		36 (26, 52)
Total anesthesia time (minutes)		64 (52, 79)
Need for general anesthesia (no/yes)		
No	1344 (94.7)	
Yes	76 (5.3)	
Time to place spinal anesthesia (minutes)		2 (1, 4)
Local anesthetic agent for spinal		
Bupivacaine (PF) 0.5% with epinephrine 1:200000	930 (65.5)	
Bupivacaine (PF) 0.5%	50 (3.5)	
Bupivacaine (PF) 0.5% with epinephrine wash	414 (29.2)	
Ropivacaine 0.5%	6 (0.4)	
Ropivacaine 0.5% with epinephrine wash	7 (0.5)	
Other	13 (0.9)	
Local spinal volume (mL)		1 (1, 1.1)
Additive used for spinal	1042 (100)	
Number of spinal attempts		
One	16 (1.1)	
Two	21 (1.5)	
Three	7 (0.5)	
Not charted	1375 (96.9)	
Was phase 1 PACU bypassed?		
No	355 (25.0)	
Yes	1065 (75.0)	
Time in phase 1 (minutes)		30 (20, 45)
Time in phase 2 (minutes)		84 (64, 128)
Total postoperative time (minutes)		82 (64, 105)
Was patient admitted?		
No	1326 (93.4)	
Yes	94 (6.6)	
Patient was admitted to inpatients ward		
Inpatient prior to procedure	28 (29.8)	
Planned admission	52 (55.3)	
Unplanned admission	14 (14.9)	
Need for supplemental oxygen intraoperatively		
No	1209 (85.1)	
Yes	211 (14.9)	
Need for additional intraoperative sedation		
No	1042 (73.4)	
Yes	378 (26.6)	

*Note:* Data presented as: Count (percentages) and median (interquartile range).

**TABLE 3:** Patients who received additional sedation intraoperatively.

Medication	*N* (%)
Dexmedetomidine	265 (18.7)
Fantanyl	19 (1.3)
Midazolam	2 (0.1)
Propofol	31 (2.2)
More than 1 sedation	61 (4.3)

*Note:* Data presented as: count (percentages).

There were few cases of intraoperative events including oxygen desaturation (3.3%), bradycardia (2%), and hypotension (17%) (Table 4). Two‐hundred and eleven patients (14.9%) received supplemental oxygen and 1 patient required the administration of an anticholinergic agent (atropine). No other intraoperative adverse events were noted. Phase I of PACU recovery was bypassed 75% of the time (Table 2).

**TABLE 4:** Intraoperative adverse effects.

Hemodynamic or respiratory problems for successful spinals	*N* (%)
SpO_2_ sustained below 92%	47 (3.3)
Heart rate < 80 beats/min	29 (2.0)
Systolic blood pressure < 60 mmHg	237 (16.7)
Administration of vasoactive agent	1 (0.1)
Patient returned to ED/UC < 7 days after procedure	56 (3.9)

*Note:* Data presented as: Count (percentages).

Abbreviations: ED, emergency department; UC, urgent care.

Fifty‐six patients (4%) returned to the hospital (Emergency Department or Urgent Care) during the first seven postoperative days. The complaints that prompted the returns were primarily related to the surgical procedure and not anesthetic care (Table 4).

## 4 Discussion

In the present manuscript, we examine the use of IT clonidine in neonates, infants, and toddlers. The cohort includes 1420 patients less than 3 years of age, and we focus specifically on the safety profile of IT clonidine. We also provide our clinical experience with the use of IT clonidine for same‐day surgery, with a large percent of our patients being discharged home. The safety profile was demonstrated as we were able to effectively collect data on readmission rates and return to the ED for perioperative concerns. Previous studies have generally focused on smaller infants with post‐surgical hospital admission.

Clonidine is an α_2_‐adrenergic agonist that was originally released in 1966 for clinical use in the treatment of hypertension. With the development of more effective medications with improved adverse effect profiles, its use for the treatment of hypertension decreased over the ensuing decades [14]. Despite this, other clinical applications have expanded to the treatment of ADHD, drug withdrawal, sleep and psychiatric disorders, and spasticity. It is available in several formulations for clinical use, including an oral tablet, transdermal patch, and a formulation for neuraxial administration [15]. Clonidine exerts its physiologic effects primarily by binding to and activating α_2_‐adrenergic receptors, resulting in sedation and analgesia. It is also known to cause potential adverse effects such as bradycardia and hypotension [16]. Previous authors have reported the use of clonidine as an adjuvant to local anesthetic agents for spinal anesthesia; these studies are outlined in Table 5 [7, 8, 11, 17, 18, 19, 20, 21, 22]. Intrathecal clonidine has been used in spinal anesthesia for both infants [4, 6, 20, 21] as well as older children [10, 15, 17, 18, 19, 20]. It has primarily been described as an adjuvant that increases spinal duration [6, 15, 17, 18], but it also can decrease overall sedation requirements [7, 10]. Here, we looked specifically at clonidine's safety profile in infants undergoing general or urologic surgeries.

**TABLE 5:** Previous reports of intrathecal clonidine use in pediatric patients.

Reference	Cohort size, age range, type of surgery	Clonidine dose (μg/kg)	Clinical outcomes
Heydinger G et al. [4]	*N* = 1221, median age 7 months; urologic and abdominal surgery	1 μg/kg	Spinal anesthesdia is viable for a number of pediatric surgical procedures with a low incidence of intraoperative and postoperative complications
Rochette A et al. [6]	*N* = 75, < 60 weeks postconceptual; inguinal herniorrhaphy	0.25, 0.5, 1, or 2 μg/kg	IT clonidine (1 μg/kg) added to IT isobaric bupivacaine doubled the duration of the blockade without significant adverse effects
Batra YK A et al. [7]	*N* = 65, 2–11 months; elective lower abdominal surgery	1 μg/kg	The requirement of propofol sedation reduces with the addition of intrathecal clonidine
Parag K et al. [10]	*N* = 42, 3–8 years; herniorrhaphy or genital surgery	1 μg/kg	IT clonidine maintained better sedation level and required less propofol than IT fentanyl as an adjuvant
Cao JP et al. [15]	*N* = 59, 6–8 years; orthopedic surgery	1 μg/kg	IT clonidine significantly prolonged the time to first rescue analgesia and reduced the requirements for propofol sedation when administered IV or IT
Gentili ME et al. [17]	*N* = 68, mean age 7 years; orthopedic surgery (clubfoot, hemiplegia, tetraplegia) and general surgery	1 μg/kg	The addition of clonidine to bupivacaine prolonged the duration of spinal anesthesia and increased the time to first dose of rescue analgesia
Kaabachi O et al. [18]	*N* = 23, 6–15 years; lower limb surgery	2 μg/kg	IT clonidine extended the duration of postoperative analgesia, but with moderate adverse effects (hypotension, bradycardia)
Kaabachi O et al. [19]	*N* = 42, 10–15 years; orthopedic surgery	1 μg/kg	IT clonidine prolinged the duration of both sensory and motor blockade and postoperative analgesia without adverse events
Rochette A et al. [20]	*N* = 124, 29–50 weeks postconceptual; herniorrhaphy	1 μg/kg	The addition of clonidine to SA in neonates results in acceptable side effects
Trifa M et al. [21]	*N* = 35, < 3 years (mean 7 months); genital, groin, and multiple site surgeries	1 μg/kg	SA (with clonidine) is a safe alternative to GA in children undergoing surgery expected to last as long as 60–100 min

We based the decision to utilize intrathecal clonidine at our institution on multiple factors. Initially, clonidine seemed a more ideal choice when compared to intrathecal opioids due to its more favorable side effect profile; clonidine is not known to cause the common opioid‐related side effects such as nausea, pruritus, constipation, and respiratory depression. Further, clonidine's ability to extend the duration of single‐shot neuraxial blockade has been well established in the pediatric literature [7]. Following the first 2 years of performing infant spinal anesthesia at our institution, we were able to publish our findings that intrathecal bupivacaine with epinephrine and clonidine, in fact, reliably produced analgesia for surgeries lasting from 60 to 100 min [21].

We have previously reported a high success rate and a low incidence of adverse effects with spinal anesthesia in infants, demonstrating its safety in the ambulatory setting in a retrospective study of patients receiving spinal anesthesia instead of GA [4]. In the present study, no patient developed apnea or bradypnea that required intervention with bag‐valve‐mask ventilation; hypoxemia was easily treated with the administration of NC oxygen, and although a low HR or sBP was noted in some patients, treatment with atropine was required only once for bradycardia. Of the 57 patients who returned to the ED/UC within 7 days after their procedure, only four patients reported respiratory distress or postoperative pain as the cause of their return, with most patients primarily citing the surgical site. Of the 14 patients that experienced unplanned admissions after surgery, only two were admitted for apnea monitoring.

Regarding the decision to utilize premedication before the procedure for anxiolysis or sedation for lumbar puncture, we do not find that this is routinely required. There is some concern that these patients are difficult to immobilize during the administration of spinal anesthesia, and other centers endorse that the routine use of premedication will improve conditions for a successful procedure [22, 23]. We find that the most important factor in the success of spinal placement is proper patient positioning and preparation of all members of the perioperative team before the patient arrives in the operating room. Education of nurses and patient families, focused anesthesiologist training, availability of supplies, optimization of surgical scheduling, and having consistent backup plans all will translate to positive clinical outcomes.

Unfortunately, our methods of data collection had limitations that might have impacted our findings and the context of their interpretation. Notably, monitor artifacts captured by the electronic medical record may have been recorded as oxygen desaturation, increasing the uncertainty of the actual incidence of hypoxemia experienced by these patients. Additionally, supplemental oxygen administration differs based on individual anesthesia provider practices and might not truly reflect the patients' need for oxygen supplementation. Another challenge to our data collection was defining the parameters of “hypotension” and “bradycardia” as our measurements of adverse events. We considered a systolic blood pressure lower than 60 mmHg to be “hypotension” and a heart rate lower than 80 beats/min to be “bradycardia”. However, because physiologic heart rate and blood pressure vary greatly within the first year of life, choosing set values can lead to inconsistent interpretation. Another limitation of our study was its retrospective nature; our cohort consisted mostly of male patients who were ASA I, which may limit the generalization of our findings to other patient populations.

Another limitation of this study is that we did not specifically collect data regarding gestational age at birth or corrected post conceptual age on the day of surgery. This will be important to explore in future prospective studies in order to determine if premature infants are more sensitive to the effects of intrathecal clonidine following spinal anesthesia. Further, an analysis comparing patient age to total sedation requirement would be enlightening and help to guide appropriate sedation strategies according to patient age.

## 5 Conclusion

Our data indicate that clonidine is a safe adjuvant in spinal anesthesia for infants presenting for outpatient ambulatory surgical procedures. The addition of clonidine maintains hemodynamic stability intraoperatively and facilitates rapid postoperative recovery. This often facilitates bypassing Phase I, resulting in a faster reunion of the patient with the family and an expeditious discharge home. Adverse effects were infrequent, mild, and easily treated.

## Conflicts of Interest

The authors declare no conflicts of interest.

## Data Availability

The data that support the findings of this study are available from the corresponding author upon reasonable request.

## References

[1] H. Fernandes , S. Santos , and A. Hazem , “Clonidine in Anesthesiology: A Brief Review,” Biomedical Journal of Science and Technical Research 7 (2018): 5815–5818.

[2] S. Crespo , G. Dangelser , and G. Haller , “Intrathecal Clonidine as an Adjuvant for Neuraxial Anaesthesia During Caesarean Delivery: A Systematic Review and Meta‐Analysis of Randomised Trials,” International Journal of Obstetric Anesthesia 32 (2017): 64–76. 28823524 10.1016/j.ijoa.2017.06.009

[3] I. Dobrydnjov , K. Axelsson , S. E. Thorn , et al., “Clonidine Combined With Small‐Dose Bupivacaine During Spinal Anesthesia for Inguinal Herniorrhaphy: A Randomized Double‐Blinded Study,” Anesthesia and Analgesia 96 (2003): 1496–1503. 12707157 10.1213/01.ANE.0000061110.62841.E9

[4] G. Heydinger , C. Roth , R. Kidwell , et al., “A Single Center's Experience With Spinal Anesthesia for Pediatric Patients Undergoing Surgical Procedures,” Journal of Pediatric Surgery 59 (2024): 1148–1153. 38418274 10.1016/j.jpedsurg.2024.02.001

[5] E. E. Whitaker , B. Z. Wiemann , D. G. DaJusta , et al., “Spinal Anesthesia for Pediatric Urological Surgery: Reducing the Theoretic Neurotoxic Effects of General Anesthesia,” Journal of Pediatric Urology 13 (2017): 396–400. 28818338 10.1016/j.jpurol.2017.06.006

[6] S. M. Walker , M. Grafe , and T. L. Yaksh , “Intrathecal Clonidine in the Neonatal Rat: Dose‐Dependent Analgesia and Evaluation of Spinal Apoptosis and Toxicity,” Anesthesia and Analgesia 115, no. 2 (2012): 450–460. 22467896 10.1213/ANE.0b013e3182501a09 PMC4455964

[7] A. Rochette , O. Raux , R. Troncin , C. Dadure , R. Verdier , and X. Capdevila , “Clonidine Prolongs Spinal Anesthesia in Newborns: A Prospective Dose‐Ranging Study,” Anesthesia and Analgesia 98 (2004): 56–59. 14693584 10.1213/01.ANE.0000093229.17729.6C

[8] Y. K. Batra , S. V. Rakesh , N. B. Panda , V. C. Lokesh , and R. Subramanyam , “Intrathecal Clonidine Decreases Propofol Sedation Requirements During Spinal Anesthesia in Infants,” Pediatric Anesthesia 20 (2010): 625–632. 20642661 10.1111/j.1460-9592.2010.03326.x

[9] M. T. Aouad, MD and R. E. Hajj, MD , “Supplementation of Intrathecal Bupivacaine With Clonidine in Ex‐Premature Neonates,” Anesthesia and Analgesia 101 (2005): 1562–1563. 10.1213/01.ANE.0000180238.73522.01 16244041

[10] T. G. Hansen and S. W. Henneberg , “Caudal Clonidine in Neonates and Small Infants and Respiratory Depression,” Pediatric Anesthesia 14 (2004): 529–530. 15153225 10.1111/j.1460-9592.2004.01313.x

[11] K. Parag , M. Sharma , H. Khandelwal , N. Anand , and N. Govil , “Intraoperative Comparison and Evaluation of Intrathecal Bupivacaine Combined With Clonidine Versus Fentanyl in Children Undergoing Hernia Repair or Genital Surgery: A Prospective, Randomized Controlled Trial,” Anesthesia, Essays and Researches 13 (2019): 323–329. 31198254 10.4103/aer.AER_24_19 PMC6545961

[12] J. A. Aldrete and D. Kroulik , “A Postanesthetic Recovery Score,” Anesthesia and Analgesia 49, no. 6 (1970): 924–934. 5534693

[13] L. Fang , Q. Wang , and Y. Xu , “Postoperative Discharge Scoring Criteria After Outpatient Anesthesia: A Review of the Literature,” Journal of Perianesthesia Nursing 38, no. 4 (2023): 642–649. 36670045 10.1016/j.jopan.2022.11.008

[14] M. J. Neil , “Clonidine: Clinical Pharmacology and Therapeutic Use in Pain Management,” Current Clinical Pharmacology 6 (2011): 280–287. 21827389 10.2174/157488411798375886

[15] H. Stähle , “A Historical Perspective: Development of Clonidine,” Best Practice & Research. Clinical Anaesthesiology 14, no. 2 (2000): 237–246.

[16] R. Aantaa , A. Marjamaki , and M. Scheinin , “Molecular Pharmacology of Alpha‐2 Adrenoceptor Agonists,” Annals of Medicine 27, no. 4 (1995): 439–449. 8519505 10.3109/07853899709002452

[17] M. E. Gentili , J. N. Ligier , J. Dermer , and J. C. Sleth , “Spinal Bupivacaine and Clonidine for Orthopaedic and General Paediatric Surgery in Remote Location,” Anaesthesia, Critical Care & Pain Medicine 36, no. 2 (2017): 131–132. 10.1016/j.accpm.2016.09.003 27671980

[18] O. Kaabachi , B. A. Rajeb , M. Mebazaa , et al., “Spinal Anaesthesia in Children: Comparative Study of Hyperbaric Bupivacaine With or Without Clonidine,” Annales Françaises d'Anesthèsie et de Rèanimation 21, no. 8 (2002): 617–621. 10.1016/s0750-7658(02)00704-9 12471781

[19] O. Kaabachi , Z. Amine , O. Rami , et al., “Clonidine 1 μg/kg is a Safe and Effective Adjuvant to Plain Bupivacaine in Spinal Anesthesia in Adolescents,” Anesthesia and Analgesia 105, no. 2 (2007): 516–519. 17646515 10.1213/01.ane.0000268709.67572.09

[20] A. Rochette , R. Troncin , O. Raux , et al., “Clonidine Added to Bupivacaine in Neonatal Spinal Anesthesia: A Prospective Comparison in 124 Preterm and Term Infants,” Pediatric Anesthesia 15, no. 12 (2005): 1072–1077. 16324026 10.1111/j.1460-9592.2005.01664.x

[21] M. Trifa , D. Tumin , E. E. Whitaker , et al., “Spinal Anesthesia for Surgery Longer Than 60 Min in Infants: Experience From the First 2 Years of a Spinal Anesthesia Program,” Journal of Anesthesia 32, no. 3 (2018): 637–640. 29808260 10.1007/s00540-018-2517-5

[22] A. Gupta and U. Saha , “Spinal Anesthesia in Children: A Review,” Journal of Anaesthesiology Clinical Pharmacology 30, no. 1 (2014): 10–18. 24574586 10.4103/0970-9185.125687 PMC3927267

[23] D. Verma , U. Naithani , C. Gokula , and n. Harsha , “Spinal Anesthesia in Infants and Children: A One Year Prospective Audit,” Anesthesia, Essays and Researches 8, no. 3 (2014): 324–329. 25886329 10.4103/0259-1162.143124 PMC4258960

